# Polyelectrolyte Multilayer Films Modification with Ag and rGO Influences Platelets Activation and Aggregate Formation under In Vitro Blood Flow

**DOI:** 10.3390/nano10050859

**Published:** 2020-04-29

**Authors:** Gabriela Imbir, Aldona Mzyk, Klaudia Trembecka-Wójciga, Ewa Jasek-Gajda, Hanna Plutecka, Romana Schirhagl, Roman Major

**Affiliations:** 1 Institute of Metallurgy and Materials Science, Polish Academy of Sciences, 25 Reymonta Street, 30-059 Krakow, Poland; aldonamzyk@googlemail.com (A.M.); k.trembecka@imim.pl (K.T.-W.); r.major@imim.pl (R.M.); 2 Department of Biomedical Engineering, University Medical Center Groningen, Antonius Deusinglaan 1, 9713AV Groningen, The Netherlands; romana.schirhagl@gmail.com; 3 Department of Histology, Jagiellonian University Medical College, 7a Kopernika Street, 31-034 Krakow, Poland; ewa.jasek@uj.edu.pl; 4 Department of Medicine, Jagiellonian University Medical College, 8 Skawinska Street, 31-066 Krakow, Poland; hanka.plutecka@uj.edu.pl

**Keywords:** polyelectrolyte multilayer films, blood-material interaction, natural heart valve, surface functionalization

## Abstract

Surface functionalization of materials to improve their hemocompatibility is a challenging problem in the field of blood-contacting devices and implants. Polyelectrolyte multilayer films (PEMs), which can mimic functions and structure of an extracellular matrix (ECM), are a promising solution to the urgent need for functional blood-contacting coatings. The properties of PEMs can be easily tuned in order to provide a scaffold with desired physico-chemical parameters. In this study chitosan/chondroitin sulfate (Chi/CS) polyelectrolyte multilayers were deposited on medical polyurethane. Afterwards PEMs were modified by chemical cross-linking and nanoparticles introduction. Coatings with variable properties were tested for their hemocompatibility in the cone-plate tester under dynamic conditions. The obtained results enable the understanding of how substrate properties modulate PEMs interaction with blood plasma proteins and the morphotic elements.

## 1. Introduction 

An integration of biomedical implants in the human organism depends on the surface properties, chemical composition, as well as mechanical properties of the materials. In the past, most clinically used materials were developed based on their acceptance by the human body. Nowadays, beneficial interactions of implant with cells and proteins gather a growing importance. An application of biomaterials in blood contacting devices is threatened by hemocompatibility failure due to the lack of an anti-thrombogenic mechanism. Under physiological conditions this mechanism is provided by the endothelium lining of the internal surface of blood vessels. An exposure to this challenging biological environment requires surface engineering for cardiovascular devices to improve the integrity with the human body [1,2,3] without influencing the structural and mechanical properties of the bulk material. Modern blood contacting materials dedicated to cardiovascular implants are expected to preferably support endothelial cells adhesion and resist interaction with blood cells that can lead to thrombosis and intimal hyperplasia [4]. Therefore, the current biomaterial development is highly related to the biocompatibility improvement through manufacturing surfaces mimicking the extracellular matrix (ECM) that will facilitate the endothelialization process in vivo. Besides providing a structural support to cells, the ECM also plays an important role in modulating numerous cellular functions. Polyelectrolyte multilayer (PEM) films made of natural polymers are one approach to obtain ECM mimicking coatings [5]. PEMs similar to the ECM, are dynamic assemblies that are undergoing remodeling in response to various stimuli, providing cells with an environment adjustable to their biochemical and mechanical properties. These may facilitate surface repopulation of the blood-contacting implant by the endothelial progenitor cells as well as mask the material from an inflammatory response and reduce risk of thrombosis in vivo. The physicochemical properties of multilayer coatings can be also modulated in vitro. Therefore PEMs are a promising system to investigate the hemocompatibility response to varying surface characteristics. The chemical cross-linking mechanism alters the interaction between polymer functional groups, therefore it is generally applied for the regulation of stability and physicochemical properties. An alternative method in PEMs modification involves nanoparticles incorporation. This new generation of modified PEMs still requires an improved understanding of how changes in their physicochemical properties regulate response of blood cells. Therefore, our work was focused on the development of PEM coatings with different characteristics. The goal was to determine material properties, which are crucial for the hemocompatibility improvement of biomaterials dedicated to cardiovascular applications. Among the natural polymers, the PEM systems composed of chitosan/chondroitin sulfate (Chi/CS) were chosen as the most promising blood-contacting materials because of efficient endothelialization [6] and potential antimicrobial effect [7]. The physico-chemical properties of the Chi/CS film were modified by chemical cross-linking or nanoparticles incorporation. In these studies, an effect of cross-linking agents N-hydroxysulphosuccinimide/1-ethyl-3-(3-dimethylaminopropyl) carbodiimide (NHS/EDC) was examined that is also widely described in literature. Two different types of nanoparticles i.e., silver (Ag) and reduced graphene oxide (rGO) have been taken into consideration because of the increasing interest in their broad spectrum of applications, especially as the antimicrobial agents in a fight against the biomaterial associated infections (BAIs) [8,9]. Moreover, it has been observed that incorporation of nanoparticles might be an alternative method of coatings stabilization [5]. The presence of nanoparticles in the film structure limits inter-diffusion of polymers and therefore has an effect on a swelling behavior and surface properties of the film. The tunable material features, developed in this work, revealed how the substrate properties modulate biomaterial hemocompatibility through the interaction with plasma proteins as well as blood-morphotic elements under dynamic conditions. 

## 2. Materials and Methods

The polyelectrolyte multilayer films were prepared from low molecular weight cationic chitosan (Chi) and anionic chondroitin sulfate (CS) purchased from Sigma-Aldrich (Saint Louis, MO, USA). Cross-linking chemicals i.e., 1-ethyl-3-(3-dimethylamino-propyl)carbodiimide (EDC) and N-hydrosulfosuccinimide (NHS) were purchased from Sigma-Aldrich. The human blood was purchased from a donation center. A plasma protein adsorption assay was performed using Qubit^®^ Protein Assay kit from ThermoFischer Scientific (Waltham, MA, USA). An impact R test kit was purchased from DiaMed Ltd. (Sofia, Bulgaria). Blood coagulation system activation was determined based on a confocal laser scanning microscopy and a flow cytometry analysis after staining with anti-CD62P FITC, anti-CD45 PE, anti-PAC-1 FITC, anti-CD61-PerCP, anti-CD14-PerCP, and anti-CD61-FITC antibodies as well as isotype controls (anti-IgG1-PE, anti-IgM-FITC, and anti-IgG1-FITC) supplied by BD Bioscience (San Jose, CA, USA). A ZymuphenMP-activity ELISA kit from Hyphen Biomed Eragny (Neuville-sur-Oise, France), was applied in a microparticles concentration analysis.

### 2.1. Polyelectrolyte Multilayer Film Build-Up 

Polyelectrolyte multilayer films (PEMs) were prepared by a “layer-by-layer” technique onto polyurethane (PU) disks (Bionate 80A, DSM Biomedical, Geleen, The Netherlands) [10]. At first, chitosan was pre-dissolved in 0.1 M acetic acid. Because of its simple solubility, the second polymer, chondroitin sulfate, was dissolved in water. No pre-dissolution step was needed. Total of 0.5 mg/mL Chi and 1 mg/mL CS were used. Polymer solutions were prepared in 0.15 M sodium chloride (NaCl) and the pH was adjusted to 5.5. Films were built-up with a special automatic dipping machine, where substrates were immersed in solutions of polycation and polyanion for 8 min each. In order to remove non-bonded polymer chains from the substrate, after the deposition process, the substrates were rinsed in 0.15 M NaCl solution set to pH 5.5. The process was repeated till the final 24 bilayer film was reached (Supplementary Information, Figure S1a).

### 2.2. PEMs Chemical Cross-Linking

The chemical cross-linking is crucial for the coating’s stability control under physiological conditions. Herein, a modification process was performed in two different approaches. In the first, the N-hydroxysulphosuccinimide (NHS) and 1-ethyl-3-(3-dimethylaminopropyl)carbodiimide (EDC) was used according to the protocol described elsewhere [11]. Chemical agents were prepared in 0.15 M NaCl solution set to pH 5.5 and then mixed together in a 1:1 volume ratio directly before incubation. Final concentrations of 260 mM EDC and 100 mM NHS were used in the experiments. Incubation of PEMs in the cross linking mixture lasted 18 h at 4 °C. Afterwards, the NHS/EDC was removed by a combination of 10 mM HEPES and 0.15 M NaCl solution buffered at pH 7.4. A two-stage washing procedure was carried out, in which films, at first, were rinsed two times for one h under slow shaking. The second stage included washing three times for 10 min (Figure S1b).

### 2.3. Nanoparticles Immobilization within PEMs

#### 2.3.1. Graphene Oxide Nanoparticles Immobilization

Graphene oxide was prepared by a modified Hummers’ method [12]. Five grams of the thermally expanded graphite and 6.5 g of potassium nitrate (KNO_3_) were mixed with highly concentrated (~96%) sulfuric acid (H_2_SO_4_). Next, 15 g of potassium permanganate (KMnO_4_) was carefully added, while the prepared solution temperature was gradually cooling down in an ice bath. The whole mixture was positioned in water at room temperature for 16 h. Once more the solution was cooled down, and afterwards deionized water was gradually poured into the mixture. Then it was heated up to 95 °C for 15 min. The process was repeated and 30% of hydrogen peroxide (H_2_O_2_) was added into the solution. Finally, the graphite oxide suspension was washed up with 3% HCl in order to remove sulfate ions. The next step involved rinsing with deionized water until none of the chloride ions were left. At the end, ultrasonification was performed for 1 h on the purified suspension [13]. Reduced graphene oxide (rGO) flakes were mixed with chondroitin sulfate (CS) in a 1:100 volume ratio and introduced into PEMs by the layer-by-layer technique (Figure S1b).

#### 2.3.2. In Situ Synthesis of Ag Nanoparticles 

Silver nanoparticles (Ag NPs) incorporation was carried out by immersing films into 0.01 mM silver salt solution (AgNO_3_) buffered at a pH around 6. The solution was allowed to incubate for 15 min, and was then washed up three times. Next, the samples were dried in an incubator at 37 °C for 30 min. Afterwards, coatings loaded with silver salt (AgNO_3_) were exposed to a 36 W UV lamp for 24 h, where the distance was settled for ~1 cm [13] (Figure S1b).

### 2.4. Coating Properties 

A static contact angle was measured by the sessile drop method with a contact angle goniometer equipped with video capture. The automatic dosing feature dispenses a water or glycerol drop (6 μL) on the coating surfaces, and the needle was manually withdrawn. An image was taken within 5 s of the placement of a drop on the surface. Contact-angle measurements were analyzed by the circle fitting profile available with the imaging software. Ten separate measurements were made on each coating at different locations.

Atomic force microscopy measurements were performed on a commercial Innova instrument. Topography pictures were obtained using tapping mode with MLCT C silicon nitride tips (Bruker). All measurements were performed at room temperature (RT). Data analysis and results presentation were realized by Nanoscope 1.40 Analysis Software. 

AFM nanoindentation measurements were carried out with 0.15 M NaCl buffered at pH 7.4. Profiles of force-indentation were performed using borosilicate sphere-tipped cantilever with a radius of 2.5 µM and a nominal spring constant of 60 mN/m. Hertz model was chosen for nanoindentation tests. This model perfectly fits the analyzed data over the indentation curve from 30 nm to 100 nm indentation depth, where the accuracy is still provided. Because of the high water content of the coatings, the incompressibility was presumed. Two measurements were performed in six different positions for each analyzed sample. The least-squares fitting method was selected for Young moduli calculations using the force-indentation curves. 

The topography of the coating with silver or graphene flakes was investigated by a scanning electron microscopy (SEM). The samples were sputter coated with a gold thin film and imaging was performed using a FEI Versa 3D FEG SEM (FEI, Krakow, Poland), with an acceleration voltage of 1 kV and electron beam current of 4.0 nA.

The deposition kinetics and hydrated film thickness were evaluated with the QCM-D technique (Q-Sense E4 system, Q-Sense AB, Gothenburg, Sweden) which has been described elsewhere [14]. The AT cut piezoelectric quartz crystals coated with gold were cleaned with piranha solution, rinsed with distilled water, dried in N2 and treated by UV prior to the experiments. The system was primed with a 0.15 M NaCl solution (buffer baseline). The polyelectrolyte solutions were injected alternately into the measurement cell at a flow rate of 50 mL/min using a peristaltic pump. A rinsing with a 0.15 M NaCl solution was performed between the adsorption of each polyelectrolyte. The thickness changes were monitored after subsequent film modification. The QCM-D method was also used to evaluate swelling of the PEM after 14 days of incubation in a physiological salt. The film thickness has been determined using the Voigt based model, defined as a spring and dashpot in parallel. The Qtools software (Q-Sense AB, Sweden) was applied to fit results. Changes in resonance frequency and dissipation for four overtones (the 3rd, 5th, 7th, and 9th overtone) were fitted.

### 2.5. Dynamic Blood-Material Interaction Assay

The Impact R test is used in clinics to evaluate thrombotic diseases and the effectiveness of anti-platelet drugs. Here this test was adjusted to detect adverse interactions between the artificial material and whole human blood. The whole blood contained sodium citrate anticoagulant and was obtained from Regional Donation Centre in Cracow, Poland. Blood was activated with adenosine diphosphate (ADP, 20 µM final concentration) for 5 min. These samples served as the control for platelets capacity to activate. Testing of materials in shear stress conditions was performed with cone-and-plate analyzer (CPA, Impact-R, DiaMed AG, Switzerland). The PU was chosen as a substrate for the PEMs deposition and as a control for our experiments because it is recently used for fabrication of cardiovascular devices. The PEMs may find future application in its surface functionalization. Polyurethane and other tested materials were prepared in the form of disks (14.4 mm diameter and 2 mm thickness). As recommended by the manufacturer, a 130 µL blood volume was used for each experiment. The dynamic test was applied at a shear rate of 1800 s for 300 s, using a disposable Teflon conical rotor. Following the shear test, the rotor was removed, and blood was immediately sampled from the well to the test tubes for flow cytometry analysis. The tested coatings were taken and evaluated by confocal laser scanning microscopy (CLSM). During the experiment the surface coverage by blood morphotic elements was determined.

### 2.6. Hemocompatibility Analysis 

#### 2.6.1. Blood Quality Analysis 

After the Impact R test, the degree of platelet activation and the number of circulating monocyte-platelet aggregates in the blood above the tested surfaces was determined. Blood was gently mixed with the following fluorochrome-conjugated monoclonal antibodies: FITC-PAC-1, PE-CD62P, and PerCP-CD61 in phosphate buffered saline (PBS) containing 0.2% bovine serum albumin and 2 mM calcium chloride. The analyses were focused on the expression of platelet markers, such as CD61, PAC-1 (antibody for conformational change of glycoprotein IIb/IIIa), and CD62P (P-selectin receptor). The platelet aggregates were counted using forward/side scatter gates for CD61 positive objects. The remaining blood plasma was separated by centrifugation at 2000× *g* for 5 min and stored frozen at −80 °C for further analysis of thrombotic activity. Thrombogenic potential of blood plasma was investigated by ZymuphenMP-activity ELISA kit, according to the manufacturer instruction [15].

#### 2.6.2. PEMs Surface Quality Evaluation

After the experiments on blood, samples were investigated for plasma proteins adsorption and surface coverage by blood morphotic elements. Plasma proteins adsorption to PEM films was estimated by the protein quantitation assay. The PEM films as well as the polyurethane (PU) control were gently rinsed by a physiological saline solution to remove all residual blood. Then samples were immersed in 1 mL of a 0.2% SDS solution and shaken for 1 h at 37 °C to release proteins adsorbed to the blood exposed surface. The protein concentration of the surface eluted samples was determined using the Qubit^®^ Protein Assay Kit and Qubit^®^ fluorometer according to producer’s instruction. The surface coverage by blood morphotic elements was analyzed using confocal laser scanning microscopy (CLSM Exciter5 AxioImager, Zeiss). In contrast, the other set of samples was gently rinsed with a physiological buffered saline solution to remove all residual blood and fixed with 1% formaldehyde. Afterwards, the specimens were stained with anti-CD62P-PE, anti-CD45-FITC, and anti-vWF- antibodies. Images were processed with the CLSM Zen 2008 software and Image-J. The contribution of blood morphotic elements, positive for the activation markers, among all cells adhered to the surface was determined based on the co-localization analysis. 

### 2.7. Statistical Investigation 

A statistical investigation (ANOVA and Tukey post hoc test, *p* value smaller than 0.05 was considered as significant—Statistica 10.0 PL) was performed on eight replicates of each sample type. The Pearson correlation was used to evaluate the relationship between the PEM physicochemical properties and hemocompatibility (*p* value smaller than 0.05 was considered as significant—OriginPro 2018).

## 3. Results

### 3.1. Coating Properties

#### 3.1.1. Surface Morphology

**Figure 1:**
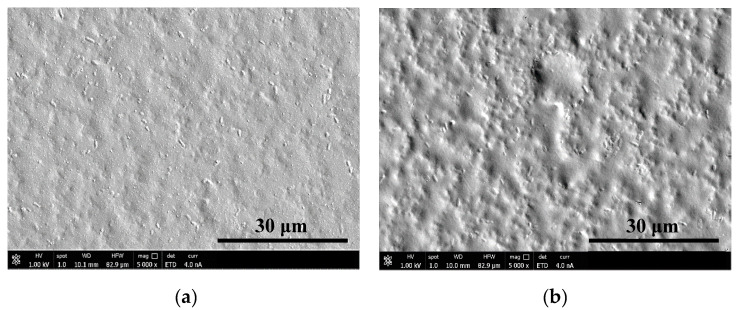
Scanning electron microscopy (SEM) images of nanoparticles distribution: (**a**) silver nanoparticles and (**b**)reduced graphene oxide flakes in chitosan/chondroitin sulfate (Chi/CS) film.

**Figure 2:**
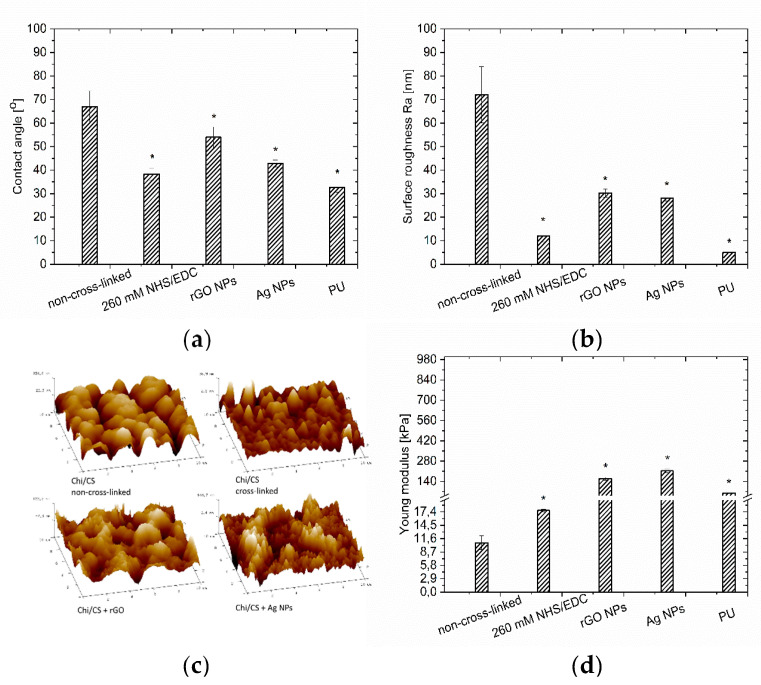
Properties of the Chi/CS films and polyurethane (PU) control: (**a**) wettability; (**b**) surface roughness; (**c**) thickness; (**d**) stiffness. Results obtained for non-cross-linked, 260 mM NHS/EDC (N-hydroxysulphosuccinimide/1-ethyl-3-(3-dimethylaminopropyl) carbodiimide) cross-linked films and samples modified with nanoparticles. The data represent mean ± SD, *n* = 8; * *p* < 0.05 vs. non-cross-linked sample.

The surface morphology of films was evaluated based on the images from scanning electron microscopy (SEM—Figure 1) and atomic force microscopy (AFM—Figure 2). The coatings completely covered the substrate surface. The Chi/CS roughness decreased after NHS/EDC treatment (12 ± 2 nm) compared to the unmodified film (72 ± 12 nm) (Figure 2b,c). The surface roughness of the PEM was determined after its structure was stabilized by nanoparticles. It has been noticed that the introduction of graphene oxide flakes (average size: 5.0 ± 2.0 nm [16]) resulted in a significant decrease of roughness (Figure 2b,c). The surface morphology of PEMs loaded with silver nanoparticles (average size: single particle 5.0 ± 1.2 nm; aggregate 40.6 ± 4.5 nm [16]) was determined. These coatings had significantly lower roughness than the unmodified films without nanoparticles (Figure 2b,c). There was significant Ra value difference between 260 mM cross-linked film and the PEM variant with silver nanoparticles. Films modified with rGO and Ag nanoparticles have comparable Ra values. The control polyurethane surface had significantly lower roughness than all analyzed coating variants. 

#### 3.1.2. Wettability 

Contact angle measurements have shown a significant difference between wettability of non-cross-linked (67 ± 7°) and modified Chi/CS films. It was found that the NHS/EDC treatment increased wettability of the Chi/CS coating (Figure 2a). The wettability of PEM was determined after nanoparticles stabilization. The wettability measurements confirmed that replacement of CS by its solution with reduced graphene oxide flakes resulted in a significant decrease in the polyelectrolyte multilayer film contact angle. Therefore its hydrophilic properties were improved (Figure 2a). The surface wettability of multilayers with silver nanoparticles nucleated by an in situ method was evaluated. Generally, the silver nanoparticles immobilization increased the contact angle value. Herein, unmodified coatings loaded with Ag had lower contact angles (43 ± 2°) than the non-cross-linked film without nanoparticles. The control polyurethane surface had significantly higher hydrophilicity than all the analyzed coating variants.

#### 3.1.3. Coating Stiffness 

The indentation test results present the mean elastic moduli of the analyzed polyelectrolyte films. It has shown the difference between elastic modulus of non-cross-linked Chi/CS and cross-linked films. Multilayer films cross-linked by NHS/EDC had significantly improved mechanical properties (Figure 2d). It was found that application of NHS/EDC resulted in higher stiffness (17.7 ± 0.3 kPa) than for the unmodified films (10.7 ± 1.5 kPa). PEMs mechanical properties were also determined after the structure stabilization by nanoparticles. It has been noticed that reduced graphene oxide flakes incorporation resulted in a significant increase of the elastic modulus value (158 ± 10 kPa) (Figure 2d). Finally, mechanical properties of PEMs with silver nanoparticles obtained by in situ nucleation were evaluated. It was found that nanoparticles immobilization led to an increase of the elastic modulus and presented the highest value among all considered groups (213 ± 12 kPa) (Figure 2d). The unmodified coating containing Ag indicated significantly higher stiffness than the unmodified film without nanoparticles. Moreover, the rigidity difference between films modified both with silver nanoparticles and graphene flakes was noticeable. 

#### 3.1.4. Coating Thickness and Stability

**Figure 3:**
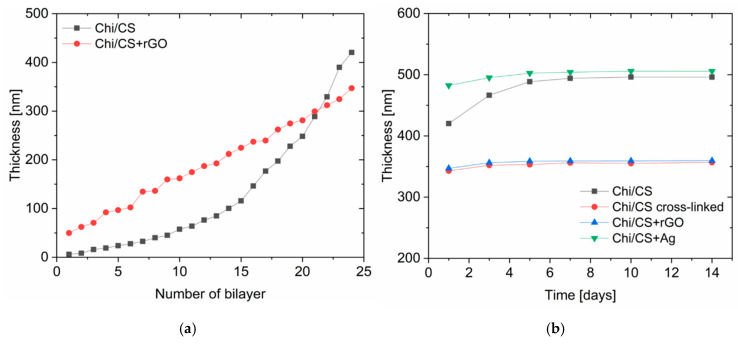
Build-up of Chi/CS multilayer film: (**a**) thickness; (**b**) swelling behavior in time.

The PEMs build-up was followed by the QCM-D (Figure 3). The Chi/CS multilayer film thickness increases exponentially as the number of bilayers increased, because of inter-diffusion of polyelectrolytes within the multilayer. The final thickness of the non-cross-linked PEM was 420 ± 15 nm. The thickness of the Chi/CS film increased to 482 ± 20 nm after in situ nucleation of silver nanoparticles. Upon cross-linking, the thickness of the native coating decreased to 343 ± 23 nm. Deposition of polyelectrolytes together with reduced graphene oxide flakes resulted in a linear build-up of the coating with the final thickness of 348 ± 12 nm. It has been found that the thickness of the non-cross-linked Chi/CS films has increased 18 ± 2% in 14 days incubation in physiological salt. The cross-linked PEMs increased their thickness of about 4.0 ± 0.6% over the time. Similar stability has been observed for films modified with nanoparticles (Ag NPs—3.6 ± 0.5%; rGO—5.0 ± 0.8%).

### 3.2. Hemocompatibility Analysis

#### 3.2.1. Blood Quality Analysis

**Figure 4:**
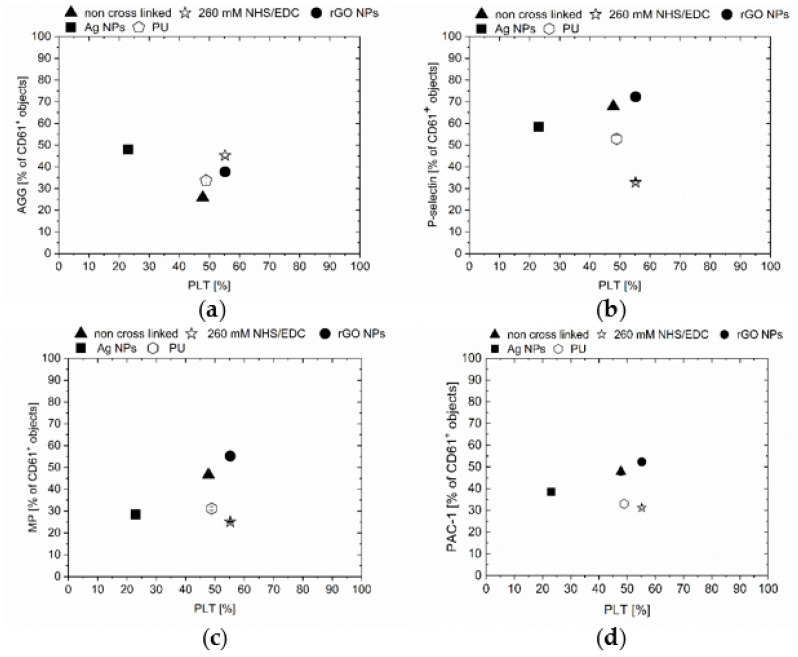
Plots ranking hemocompatibility of Chi/CS films: (**a**) Percentage of platelet aggregates (AGG); (**b**) percentage of P-selectin positive platelets (P-SEL); (**c**) concentration of microparticles thrombogenic activity (MP); (**d**) percentage of PAC-1 positive platelets (PAC-1). All values are plotted against the percentage of platelets that remained after the shear stress (PLT). The data represent mean ± SD, *n* = 8.

The blood quality analysis indicated a significant difference between Chi/CS unmodified and cross-linked films (Figure 4). Chi/CS in the native state caused significantly higher platelet activation (Figure 4b,c) and correlated contribution of microparticles than the polyurethane samples. The chemical cross-linking (by NHS/EDC) was responsible for an increase in platelet aggregates formation and decrease of P-selectin and PAC-1 positive platelets. Additionally, we observed more microparticles compared to the non-cross-linked multilayers (Figure 4). The hemocompatibility of the Chi/CS films was determined after its structure stabilization by nanoparticles. It has been noticed that reduced graphene oxide flakes introduction resulted in a significant increase of platelets activation and microparticles contribution in comparison to both control sample and the unmodified coating. It also led to erythrocytes hemolysis. Blood quality changes were also determined after contact with PEMs containing silver nanoparticles. The silver nanoparticles immobilization led to a decrease of platelets activation and microparticles contribution when compared to the non-cross-linked coatings. Simultaneously, the blood quality analysis indicated a higher number of aggregates.

#### 3.2.2. PEMs Surface Quality Evaluation 

**Figure 5:**
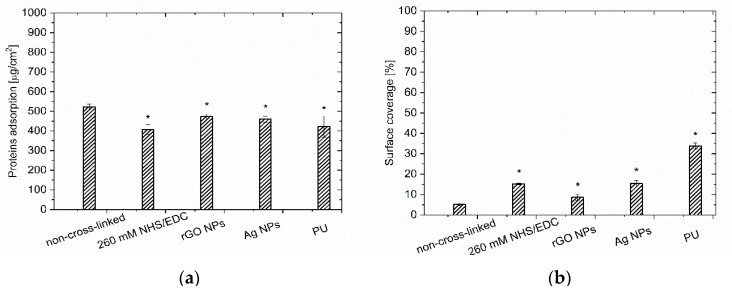
(**a**) Amount of adsorbed proteins and its effect on (**b**) surface coverage of Chi/CS film unmodified, cross-linked with 260 mM NHS/EDC and modified by reduced graphene oxide flakes and silver nanoparticles by blood morphotic elements. Data represent mean ± SD, *n* = 8; * *p* < 0.05 vs. non-cross-linked sample.

Parallel to the blood quality determination, the hemocompatibility analysis concerned the samples’ surface investigation. Plasma proteins adsorption to PEM films was estimated by a protein quantitation assay. Herein, it has been shown that unmodified Chi/CS caused similar protein adsorption as the polyurethane samples (Figure 5a). The chemical cross-linking was responsible for a decrease in protein adsorption compared to the non-cross-linked multilayer. It was found that the application of NHS/EDC resulted in a lower protein adsorption than on the unmodified film. Surface coverage by plasma proteins was also determined after Chi/CS film structure stabilization by nanoparticles. It has been observed that the introduction of the reduced graphene oxide flakes resulted in a significant decrease in protein adsorption compared to the native coatings. Similarly, the silver nanoparticles immobilization within PEM led to a decrease of adsorbed proteins on the coating surface. 

**Figure 6:**
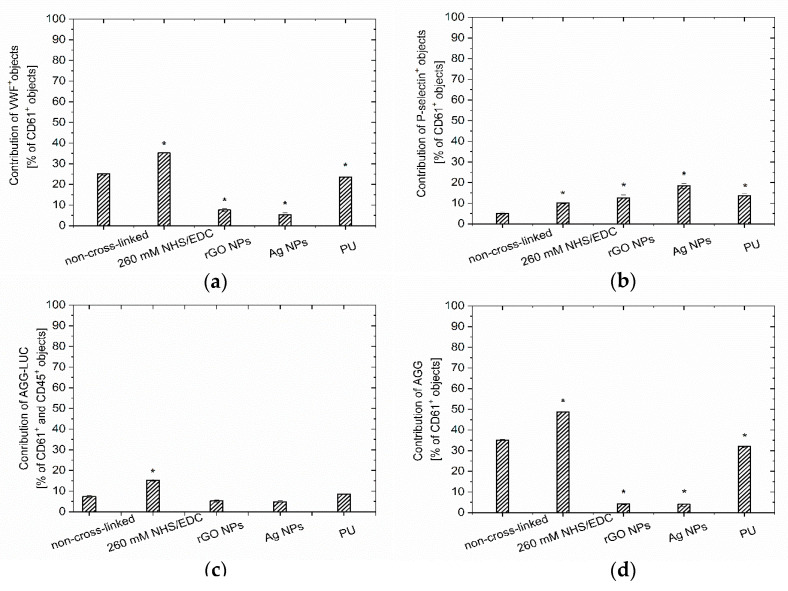
The contribution of (**a**) vWF positive objects; (**b**) P-selectin positive platelets; (**c**) AGG-LUC platelet-leukocyte aggregates, and (**d**) AGG platelet aggregates on the surface of unmodified Chi/CS film, cross-linked with 260 mM NHS/EDC and modified by graphene flakes and silver nanoparticles. Data represent mean ± SD, *n* = 8; * *p* < 0.05 vs. non-cross-linked sample.

The blood–material interactions were evaluated based on the tests simulating aortic-shear-stress conditions. The microscopic observations of the sample’s surface were performed in order to assess the number of aggregates (leukocyte-platelet, platelet-platelet, monocyte-platelet), activated platelets and adhered leukocytes (Figure 5b and Figure 6). The unmodified Chi/CS surface observations indicated the lowest coverage by blood elements among all the investigated samples (including PU control). Chemical cross-linking of the Chi/CS film with NHS/EDC solution increased the surface coverage, however the obtained values were below the ones for the control sample. The cross-linking of the Chi/CS coating increased the level of the von Willebrand’s factor, P-selectin positive objects as well as the contribution of both platelet and platelet-leukocyte aggregates (Figure 6). The hemocompatibility was also determined for PEMs with nanoparticles. It has been noticed that the immobilization of the reduced graphene oxide flakes resulted in a lower blood element adhesion on tested coatings when compare to PU control and the NHS/EDC cross-linked film. However, we observed a higher surface coverage than on the native Chi/CS. In the presence of rGO, the level of the von Willebrand’s factor and platelet aggregates decreased, whereas the number of P-selectin positive objects was significantly higher than on the unmodified PEMs. Blood—material interaction was also determined for the film containing silver nanoparticles. Surface investigation has shown higher surface coverage, especially with the P-selectin positive objects than in case of the native Chi/CS film. The level of the von Willebrand’s factor and platelet aggregates decreased in comparison to unmodified as well as cross-linked films. 

The number of blood elements on the surface of each type of investigated PEM was lower than in case of the PU sample. There was no significant difference between coatings modified by reduced graphene oxide flakes and silver nanoparticles. 

### 3.3. Correlation of PEMs Physicochemical Properties with Hemocompatibility

The relationship between physicochemical properties of the PEMs (roughness, wettability, and stiffness) and the hemocompatibility parameters was determined based on the Pearson correlation analysis (Table 1). It has been found that the proteins adsorption is positively correlated with surface roughness and contact angle. The expression of P-selectin has a strong positive correlation with the film stiffness and a weak negative correlation with the surface roughness and wettability. The performed evaluations have shown that a number of vWF positive objects and platelet-based aggregates is negatively correlated with coatings stiffness. The formation of platelet-monocyte aggregates was independent of the surface properties. The evaluation of blood parameters correlation with surface properties has shown that the coatings stiffness had an influence only on the formation of the platelet-based aggregates. The number of P-selectin positive objects in blood had weak positive correlation with surface roughness and wettability. The strong correlation has been found between contact angle and the number of microparticles as well as the PAC-1 positive objects. 

## 4. Discussion

### 4.1. Simultaneous Multiparameter Changes in the Physicochemical Properties of PEMs 

The PEM films differ with the internal structure, and the surface properties depend on if they are built from weak or strong polyelectrolytes, or a combination of the above mentioned. In this study, PEMs were fabricated based on an interaction between chitosan, which is a week polycation with a strong polyanion, chondroitin sulfate. The strong polyanion presence in the PEM made it thin and hydrophobic because of the increased electrostatic complexation with chitosan counterions. Chitosan has a high charge density especially in acidic environments, which allowed a formation of ionic complexes and acted as a driving force in coating formation. Interaction of hydrogen bonds defined the hydration of chondroitin sulfate, which had an effect on film thickness. Fu et al. discovered changes in PEM roughness with the increase of pH solution in chitosan/heparin coatings. PEMs surfaces were smoother if the polyelectrolytes were deposited from strong acidic solutions (~pH 4). Chitosan possesses weak chain charges at higher pH values. Therefore, films had a rougher surface when assembled at high pH. Similarly, wettability results showed high contact angle in films assembled at pH 6, whereas smallest contact angle, which indicates hydrophilicity, was found at pH 3 [17]. Crouzier et al. have observed that the pH and ionic strength also determined mechanical properties of PEMs. The stiffness of the polysaccharide–polysaccharide system was dependent on exponential film build up [18]. These films were considered to be softer than PEMs, which are built up in linear manner. In this study, the Chi/CS multilayers have been deposited from the solutions of pH 5.5. The investigated unmodified PEM has shown exponential thickness growth. Oppositely, to the previously described chitosan/heparin films, our coatings were rougher and stiffer. Moreover, it has been found that the films were more hydrophobic than other polysaccharide-based PEMs deposited under similar conditions [17]. 

Further changes in surface roughness, wettability, and stiffness of Chi/CS films were observed after the applied cross-linking. Coatings became smoother, stiffer, and more hydrophilic in comparison to the native ones. We attribute the changes to new bond formation between free functional groups and a lower level of inter-diffusion within the coating. Schneinder et al. have also proven that cross-linked PEMs were ten times stiffer than native films. They have shown that the degree of cross-linking influenced film structure. It led to an increase in roughness and hydrophilicity [11,19].

Incorporation of nanoparticles in the PEMs was used as an alternative method for stabilization of film structure, but also in order to modulate its physicochemical properties and provide with antimicrobial potential. Over the years, various methods for introduction of metal nanoparticles in PEMs have been developed. One of the approaches is in situ photoreduction, in which films can be immersed in AgNO_3_ solution and then exposed to UV radiation. While the ionized acid groups, e.g., carboxylic acid and sulfuric acid groups are responsible for electrostatic interaction with the polycation, the nonionized acid groups can bind to metal ions via ions exchange [20]. The reduction method chosen for the in situ process could have an effect on the surface properties. Jang et al. have observed that the presence of AgNPs in the structure of poly(ethyleneimine)/hyaluronic acid multilayers led to an increase in roughness. The same researchers have described the relation between the number of bilayers and AgNP formation [13]. Mzyk et al. analyzed PEMs structure of chitosan/chondroitin sulfate and have found that AgNP did not create a separate layer within the multilayer, but it was distributed in PEMs in the form of aggregates [16]. Moreover, they have found that stiffness of AgNP-PEMs was significantly higher than in the case of unmodified films. Wettability investigation of strong polyelectrolytes interaction presented by Zan et al. indicated ion exchange of counterions Na^+^ and Ag^+^, in which the ion pair R-SO_3_-Ag^+^ was less hydrated than R-SO_3_-Na^+^. This resulted in higher hydrophobicity of the silver containing PEM [21]. In this article, we have observed that introduction of silver nanoparticles led to a decrease in surface roughness, differently to results presented by Jang et al. [13]. Wettability analysis showed an increase of the hydrophilicity of AgNP films. The coatings also demonstrated higher stiffness in comparison to unmodified films. Hence, the formation of polyelectrolyte-silver complexes resulted in a certain distribution of silver nanoparticles which affected the surface properties. 

Besides the modification with the silver nanoparticles, our work also presents another approach to modulate PEM’s physicochemistry. rGO flakes were mixed with a polyanionic solution and introduced into the film during the layer-by-layer deposition process. Graphene oxide (GO) indicates amphiphilic properties, where the basal plane is hydrophobic, and edges are hydrophilic [22]. Because of multiple oxygen-containing groups i.e., hydroxyl, carboxyl, reduced graphene oxide particles can create strong interactions with chondroitin sulfate (polyanion), such as hydrogen or π–π bonding and can be easily dispersed [23]. Thus, in this study reduced graphene oxide immobilization within PEM caused an increase in stiffness and hydrophilicity. According to Kulkarni et al. the surface roughness of PEMs after modification with rGO decreases because of the atomic smoothness of rGO [24]. In the presented work, an analogous phenomenon was observed. Hence, regarding the morphological properties of PEMs incorporated with silver nanoparticles and reduced graphene oxide, it was found that both systems indicate similar physicochemical properties. 

### 4.2. Correlation of the PEMs Physicochemical Properties with Hemocompatibility

The success of an implanted biomaterial depends on the biological reactions occurring at its surface. Protein adsorption from blood is recognized as the first event following blood–biomaterial contact that depends on the previously elaborated surface properties such as chemistry, wettability, stiffness, and topography [25]. The adsorbed protein layer may subsequently influence the platelet activation and aggregation process. Therefore, an understanding of the interactions between implants and the surrounding biological environment has been a major focus of this research. 

The influence of biomaterial roughness on thrombogenicity is not clear, and various studies have shown conflicting results. Cai et al. [26] reported that the surface roughness had a little effect on protein adsorption on titanium materials with roughness values in the range of 2–21 nm. When surface features are in the same size regime as the adsorbing protein, geometric parameters such as height, width, and the separation of the nanofeatures are likely to affect how the proteins adsorb as proposed by Roach et al. They have suggested that a high aspect ratio and small separation (<200 nm) of nano features significantly reduced platelet activation [25]. It was also found that adsorption of the platelet-binding protein fibrinogen was reduced on surface features in the same size regime. In an earlier publication they found that surface nanostructures in the sub 100 nm regime attenuated the activation of the immune complement system. They proposed a mechanism where protein–surface interactions were altered because of the curvature of the nanostructure. Bailly et al. compared angiographic catheters with different surface roughness and concluded that the smoothest material was thrombogenic [27]. Zingg et al. described that the increased roughness under dynamic conditions caused a decrease in platelet adhesion on hydrophilic surfaces and opposite response on hydrophobic surfaces. During the static test conditions, no difference between smooth and rough surfaces was found [28]. In this study, it has been found that PEM’s morphological properties affected proteins adsorption. Herein, rougher surface have promoted higher protein adsorption. The unmodified films indicated the worst antiadhesive properties. Although, we have observed changes in the amount of adsorbed proteins, these changes did not correlate with the level of P-selectin objects, the von Willebrand factor and the number of aggregates attached to the PEM surface. Our research clearly shows that PEM’s topography has an influence on the platelet’s activation. However, we have not found that obvious correlation of the film roughness with the number of aggregates and von Willebrand factor on its surface. Moreover, we have not observed correlation of the number of platelet aggregates in blood with the roughness of the investigated PEM. A weak correlation with film surface morphology was found for the activated platelets and the microparticles in blood, for which the number increased after contact with rougher coatings.

While the thrombus adhesion seems to be related to surface texture, some researchers have shown that the formation of a thrombus depends on the wettability of biomaterial. Contact angles above the 65° can have a significant impact on proteins adsorption, in a manner that depends on the nature of the functional groups. Wettability influences the time-dependent conformational changes in adsorbed proteins and mediate adsorption kinetics and binding strengths [29], as well as subsequent protein activity [30,31]. Results of plasma protein adsorption presented in this study agree with the previous work [32], where protein adsorption increased on surface with higher hydrophobicity. Moreover, this research has shown that an increase in wettability was positively correlated with changes of coating hemocompatibility under dynamic conditions. Platelets that adhered to the hydrophilic PEM are easily detached from the surface under applied shear forces and less likely formed aggregates than on hydrophobic surface. In our previous work we have shown that the described effect is related to higher contribution of the von Willebrand factor in aggregate formation when blood gets into the contact with the hydrophilic surface [32]. In this study we have also observed the higher number of circulating aggregates in the presence of hydrophilic coatings that was correlated with a lower contribution of the P-selectin positive platelets. 

How the mechanical properties of the PEMs affect platelet activation and physiology under blood flow, also remains largely unknown. Oui Y. et al. [33] found that the number of adherent platelets on the PA gel surfaces after incubation increased with increasing substrate stiffness, reaching a plateau (~10,000 platelets per mm^2^) on 50-kPa PA gels and stiffer. Moreover, their findings suggest that fibrinogen concentration and substrate stiffness are independent factors that affect platelet adhesion and spreading and appear to affect platelet behavior synergistically. But the relationship between the effects may not be linear. This is consistent with the report showing that platelet adhesion and spreading on immobilized fibrinogen involve different signaling pathways [34]. Yu D. et al. have shown that platelet adhesion had increased with the number of PEM layers and suggested that the swelling or mechanical properties of the PEM films may not direct the platelet adhesion [35]. Kerch G. et al. designed PEMs based on chitosan/chitosan sulfate and described that stiffness of the outermost layer is important to determine the platelets adhesion—if the stiffness is low, the adhesion decreases [36]. In this study, we have not observed a relation of coating stiffness with the amount of adsorbed proteins. However, we have found that the number of activated platelets (the P-selectin positive) on the surface increased with increasing the coating stiffness. In contrary, there were less aggregates and von Willebrand factor found on the stiffer films. We also found a significant increase in the number of aggregates circulating in the blood after contact with stiffer PEMs. 

Although this work already elaborates the influence of multiple physicochemical parameters on hemocompatibility, we have realized that even broader characterization is necessary for further understanding of the phenomena. We have found that the investigated physicochemical properties of films modified with silver and reduced graphene oxide were similar. In our opinion, the discrepancy in the hemocompatibility of these PEMs may be a result of the different type of functional groups exposed on the film surface or/and their electrokinetic potentials. Meng et al. have described the chitosan/heparin films interaction with blood under dynamic conditions [37]. They discovered that platelets easily attached to weak polyelectrolytes such as chitosan. On the other hand, heparin (Hep), which is a strong polyelectrolyte, proved to inhibit adhesion, therefore the whole system indicated anticoagulation properties. Authors have suggested that the improved hemocompatibility was a result of the electrostatic repulsive interaction between negatively charged functional groups of heparin, proteins, and platelets. Similarly, Kuo et al. have analyzed blood-PEMs interaction under static conditions and they confirmed that once the outermost layer in PEMs is negatively charged then the adhesion of platelets is significantly lower [38]. Research presented by Kerch et al. showed lower platelet adhesion for chitosan/heparin films, where chitosan was deposited as an outermost layer. This research led to the conclusion that the surface charge is not the key regulator of platelet adhesion [36]. In this study, negatively charged chondroitin sulfate was deposited as the outermost layer. In comparison to heparin, which possesses strong anticoagulant properties, the CS has a smaller amount of sulfate groups. This may have a strong influence on the PEM wettability and therefore results in different response from the blood morphotic elements. Studies of other authors on self-assembled monolayers (SAMs) functionalized with controlled moieties showed that after whole blood incubation, surfaces functionalized with –CH_3_ groups lead to an increased platelet adhesion, while on –OH terminated SAMs only leukocytes adhere. In contrast neither platelets nor leukocytes adhere on –COOH presenting surfaces [39]. Herein, coatings with incorporated silver and reduced graphene oxide flakes showed lower levels of platelet adhesion, which may be related to the higher number of negatively charged groups on the surface compared to the unmodified films. –OH groups on the surface of coatings with rGO were found by many researchers. However, contrary to the previously discussed research, herein we have not observed increase of leucocytes attachment. It could be an effect of their lower availability due to compensation by positively charged groups from chitosan. It is also possible that functional groups other than –OH were more abundant at the surface of graphene flakes. In a consequence this may lead to the formation of a protein layer which vary in a composition and/or conformation of molecules and eventually result in different blood-material interactions. This hypothesis should be the subject of further investigations.

## 5. Conclusions

A simple and universal method is still needed for surface modification to improve the hemocompatibility of biomaterials. In this work we have presented, for the first time, how the PEM’s physicochemical properties influence blood proteins adhesion, platelets activation, and aggregates formation under dynamic conditions. At the same time PEMs, as an easily tunable film model, are giving the opportunity to find relations between properties which may also apply to many other complex coatings designed for the functionalization of cardiovascular devices. We have found strong correlation between stiffness of the PEMs and active platelets and platelet-based aggregates adhesion to the surface. Moreover, based on the obtained results we think that wettability determines attachment stability of the active platelets and aggregates to the PEMs. In comparison to our previous work on hemocompatibility of the carbon-based thin ceramic coatings, the phenomena observed at the blood—PEMs interface could not be described easily either by rolling, or two-stage aggregation mechanism. It will be possible only by further developing the understanding of cross-talk between various surface parameters. 

## Figures and Tables

**Table 1:** Correlation of the Polyelectrolyte multilayer (PEMs) physicochemical properties with hemocompatibility. Colors indicate correlation strength: green—strong correlation; yellow—weak correlation; red—no correlation.

Hemocompatibility Parameter		PEM Properties		
		Roughness	Contact Angle	Stiffness
Surface	Protein adsorption	**0.95**	**0.94**	**0.05**
	P-selectin expression	**−0.61**	**−0.65**	**0.91**
	vWF contribution	**−0.09**	**−0.15**	**−0.94**
	Platelet-monocytes aggregates	**−0.32**	**−0.08**	**−0.24**
	Platelet aggregates	**−0.06**	**−0.14**	**−0.94**
Blood	P-selectin expression	**0.62**	**0.69**	**0.40**
	PAC-1 expression	**0.60**	**0.80**	**0.20**
	Platelet aggregates	**−0.28**	**−0.11**	**−0.64**
	Microparticles	**0.58**	**0.77**	**0.10**

## Supplementary Materials

The following are available online at https://www.mdpi.com/2079-4991/10/5/859/s1, Figure S1: Layer-by-layer deposition of the chitosan/chondroitin sulfate (Chi/CS) polyelectrolyte multilayer film: (a) unmodified PEMs, (b) PEMs modification by cross-linking with NHS/EDC, *in situ* synthesis of silver nanoparticles and incorporation of reduced graphene oxide flakes.
