# Supplementary material for: Polyelectrolyte Multilayer Films Modification with Ag and rGO Influences Platelets Activation and Aggregate Formation under In Vitro Blood Flow
Source: Nanomaterials (Basel). 2020 Apr 29;10(5):859. doi: 10.3390/nano10050859 (PMC7712484; doi:10.3390/nano10050859)
Supplement: Supplementary file 1 [file nanomaterials-10-00859-s001.pdf]

Supplementary Materials: Polyelectrolyte Multilayer Films Modification with Ag and rGO Influences Platelets Activation and Aggregate Formation under In Vitro Blood Flow

Gabriela Imbir, Aldona Mzyk, Klaudia Trembecka-Wójciga, Ewa Jasek-Gajda, Hanna Plutecka, Romana Schirhagl and Roman Major

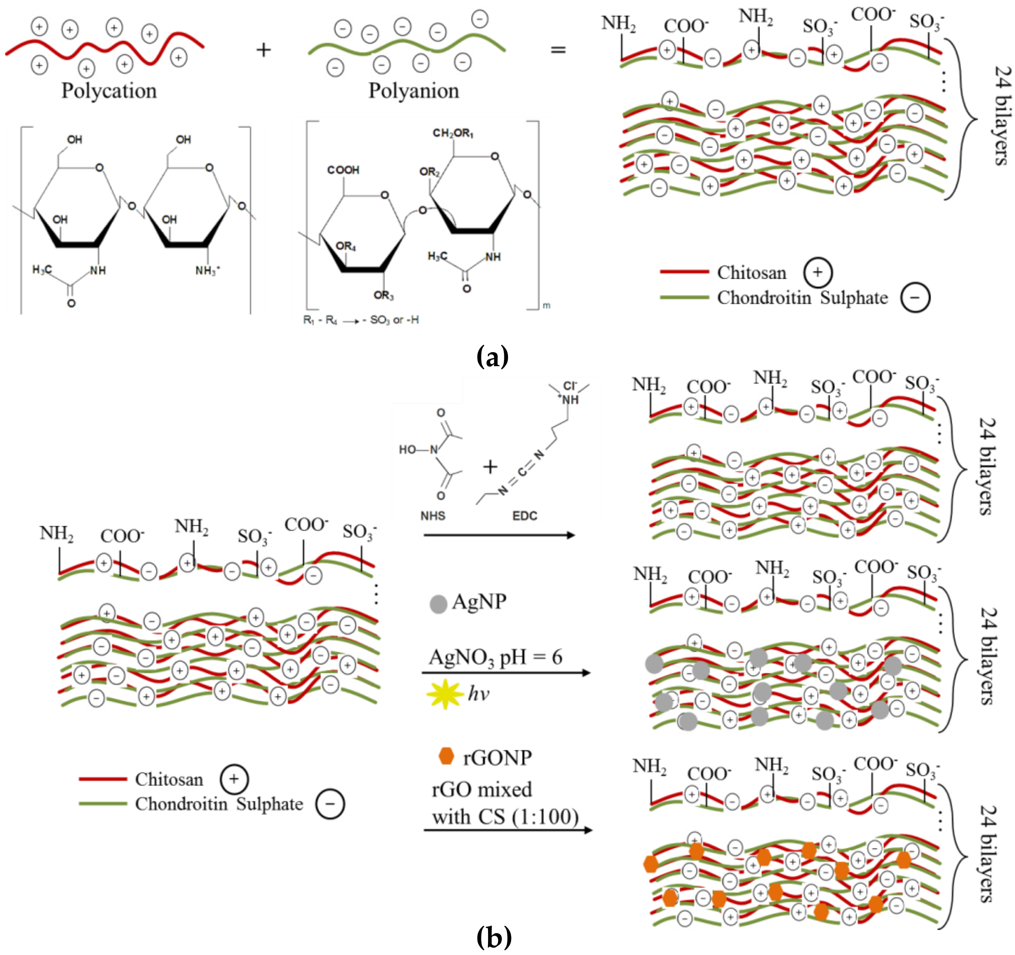

Figure S1. Layer-by-layer deposition of the chitosan/chondroitin sulfate (Chi/CS) polyelectrolyte multilayer film: (**a**) unmodified PEMs, (**b**) PEMs modification by cross-linking with NHS/EDC, *in situ* synthesis of silver nanoparticles and incorporation of reduced graphene oxide flakes.
